# Supplementary material for: Popcorn (Zea mays L. var. Everta) haploids identified by Navajo phenotype and ploidy level
Source: Front Plant Sci. 2023 May 30;14:1176504. doi: 10.3389/fpls.2023.1176504 (PMC10266278; doi:10.3389/fpls.2023.1176504)
Supplement: Supplementary file 1 [file DataSheet_1.docx]

Supplementary Material

Popcorn haploids identified by Navajo phenotype and ploidy level

Jean Paulo Aparecido da Silva*, José Marcelo Soriano Viana, Kaio Olimpio das Graças Dias, Jéssica Coutinho Silva, Vivian Torres Bandeira Tupper, Wellington Ronildo Clarindo

* Correspondence: [jean.p.silva@ufv.br](mailto:jean.p.silva@ufv.br)


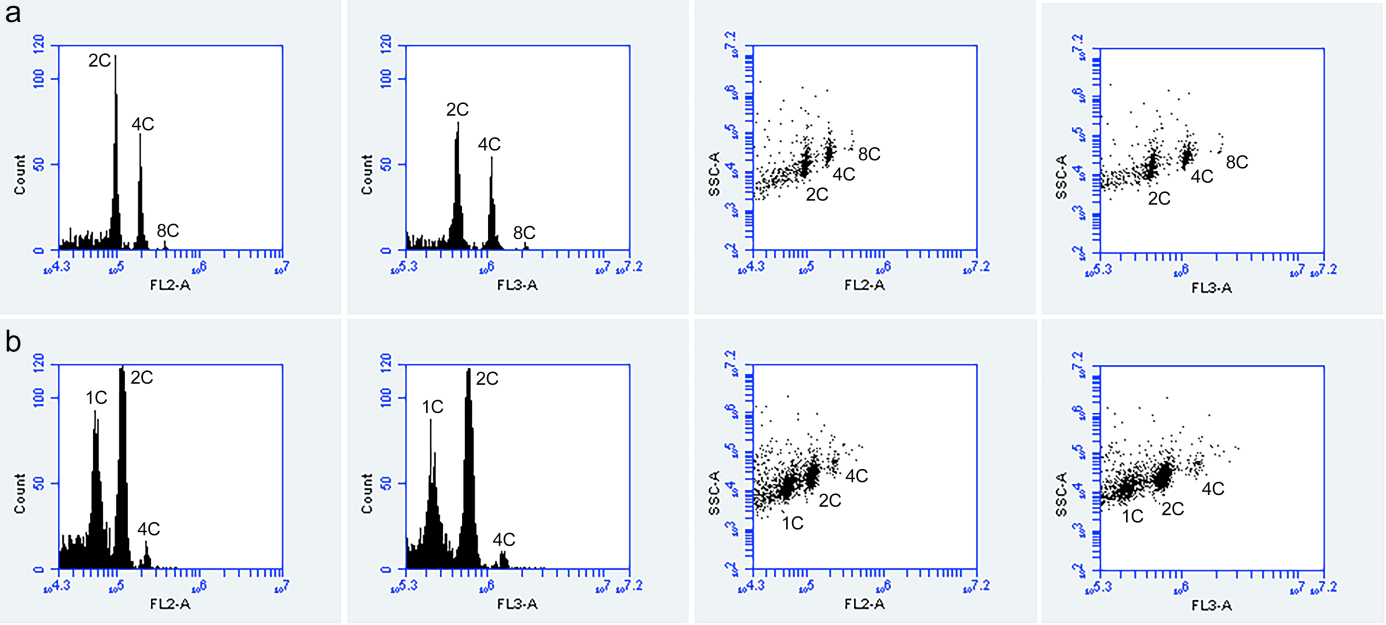


**Supplementary Figure 1.** Representative flow cytometry histograms of the (**a**) diploid and (**b**) induced haploid *Z. mays* popcorn plants. For the same nuclei suspension, the propidium iodide fluorescence of the nuclei was detected using the FL2 (615 — 670 nm) and FL3 (> 670 nm) filters, and monoparametric (nuclei count *vs* propidium iodide fluorescence) and biparametric (nuclei count *vs* propidium iodide fluorescence *vs* SSC) histograms.  The diploid *Z. mays* popcorn showed 2C, 4C and 8C nuclei, while induced haploid *Z. mays* popcorn exhibited 1C, 2C and 4C nuclei, confirming the ploidy level of these plants.

**Supplementary Table 1.** Description of treatments.

| Germplasm | Genetic basis | Origin | Type |
| --- | --- | --- | --- |
| 19-1206-6 | Inbred line | Temperate | Popcorn |
| 19-1504-4 | Inbred line | Temperate | Popcorn |
| 18-390-1 | Inbred line | Temperate | Popcorn |
| 18-303-2 | Inbred line | Temperate | Popcorn |
| 20-2039 | Inbred line | Tropical | Popcorn |
| 18-176-5 | Inbred line | Temperate | Popcorn |
| 20-2009 | Inbred line | Tropical | Popcorn |
| 20-2010 | Inbred line | Tropical | Popcorn |
| 20-2064 | Inbred line | Tropical | Popcorn |
| 20-2024 | Inbred line | Tropical | Popcorn |
| UFV-MP1 | Population | Temperate | Popcorn |
| UFV-MP2 | Population | Temperate | Popcorn |
| UFV-MP3 | Population | Temperate | Popcorn |
| UFV-MP4 | Population | Temperate | Popcorn |
| UFV-MP5 | Population | Temperate | Popcorn |
| Viçosa C4 | Population | Tropical | Popcorn |
| Beija-Flor C4 | Population | Tropical | Popcorn |
| Synthetic UFV | Population | Tropical | Popcorn |
| Viçosa C4 x Beija Flor C4 | Interpopulation hybrid | Tropical | Popcorn |
| Beija-Flor C4 x Synthetic UFV | Interpopulation hybrid | Tropical | Popcorn |
| UFV-MP2 x UFV-MP5 | Interpopulation hybrid | Temperate | Popcorn |
| Viçosa-C4 x UFV-MP2 | Interpopulation hybrid | Tropical x Temperate | Popcorn |
| Viçosa C4 x UFV-MP4 | Interpopulation hybrid | Tropical x Temperate | Popcorn |
| Viçosa C4 x UFV-MP5 | Interpopulation hybrid | Tropical x Temperate | Popcorn |
| Synthetic UFV x UFV-MP1 | Interpopulation hybrid | Tropical x Temperate | Popcorn |
| 2B710PW | Single-cross | Tropical | Maize |
| MG580PW | Single-cross | Tropical | Maize |
| ES481PW | Single-cross | Tropical | Maize |
| Feroz | Two-way cross | Tropical | Maize |
| AGS Lavrador OP | Population | Tropical | Maize |

**Supplementary Table 2.** Screening by the Navajo phenotype and haploid induction rate (HIR).

| Germplasm | Ear | Haploid | Diploid | Yellow | Purple | Total | HIR |
| --- | --- | --- | --- | --- | --- | --- | --- |
| 19-1206-6 | - | - | - | - | - | - | - |
| 19-1504-4 | - | - | - | - | - | - | - |
| 18-390-1 | - | - | - | - | - | - | - |
| 18-303-2 | - | - | - | - | - | - | - |
| 20-2039 | - | - | - | - | - | - | - |
| 18-176-5 | 15 | 141 | 903 | 1553 | 1044 | 2597 | 5.44 |
| 20-2009 | 6 | 9 | 614 | 9 | 623 | 632 | 1.41 |
| 20-2010 | 14 | 63 | 3060 | 209 | 3123 | 3332 | 1.95 |
| 20-2064 | 11 | 30 | 1039 | 171 | 1069 | 1240 | 2.38 |
| 20-2024 | 7 | 29 | 966 | 168 | 995 | 1163 | 2.37 |
| UFV-MP1 | 9 | 4 | 736 | 165 | 740 | 905 | 0.45 |
| UFV-MP2 | 27 | 12 | 2066 | 577 | 2078 | 2655 | 0.45 |
| UFV-MP3 | 15 | 15 | 623 | 46 | 638 | 684 | 2.14 |
| UFV-MP4 | 14 | 3 | 334 | 108 | 337 | 445 | 2.27 |
| UFV-MP5 | 21 | 5 | 634 | 236 | 639 | 875 | 0.86 |
| Viçosa C4 | 75 | 54 | 2203 | 2180 | 2257 | 4437 | 1.23 |
| Beija-Flor C4 | 41 | 7 | 232 | 540 | 239 | 779 | 1.07 |
| Synthetic UFV | 99 | 252 | 11169 | 6047 | 11421 | 17468 | 1.41 |
| Viçosa C4 x Beija Flor C4 | 64 | 9 | 1067 | 2088 | 1076 | 3164 | 0.33 |
| Beija-Flor C4 x Synthetic UFV | 29 | 8 | 1047 | 749 | 1055 | 1804 | 0.45 |
| UFV-MP2 x UFV-MP5 | 24 | 22 | 1009 | 314 | 1031 | 1345 | 2.25 |
| Viçosa-C4 x UFV-MP2 | 31 | 9 | 1573 | 1097 | 1582 | 2679 | 0.34 |
| Viçosa C4 x UFV-MP4 | 28 | 21 | 6124 | 1836 | 6145 | 7981 | 0.37 |
| Viçosa C4 x UFV-MP5 | 44 | 4 | 2290 | 1414 | 2294 | 3708 | 0.11 |
| Synthetic UFV x UFV-MP1 | 77 | 46 | 3410 | 1883 | 3456 | 5339 | 0.86 |
| 2B710PW | 110 | 59 | 49945 | 9610 | 50004 | 59614 | 0.10 |
| MG580PW | 118 | 63 | 48055 | 9597 | 48118 | 57715 | 0.11 |
| ES481PW | 124 | 20 | 27173 | 18483 | 27193 | 45676 | 0.04 |
| Feroz | 34 | 22 | 10300 | 2968 | 10322 | 13290 | 0.17 |
| AGS Lavrador OP | 57 | 10 | 21053 | 1277 | 21063 | 22340 | 0.05 |
| Total | 1094 | 917 | 197625 | 63325 | 198542 | 261867 | - |

**Supplementary Table 3.** Screening by the seedling vigor.

| Germplasm | Seedlings | Haploids | False positive |
| --- | --- | --- | --- |
| 18-176-5 | 108 | 37 | 71 |
| 20-2009 | 9 | 9 | 0 |
| 20-2010 | 63 | 44 | 19 |
| 20-2064 | 30 | 24 | 6 |
| 20-2024 | 29 | 22 | 7 |
| UFV-MP1 | 4 | 4 | 0 |
| UFV-MP2 | 12 | 8 | 4 |
| UFV-MP3 | 15 | 12 | 3 |
| UFV-MP4 | 3 | 3 | 0 |
| UFV-MP5 | 5 | 4 | 1 |
| Viçosa C4 | 54 | 32 | 22 |
| Beija-Flor C4 | 7 | 3 | 4 |
| Synthetic UFV | 54 | 33 | 21 |
| Viçosa C4 x Beija Flor C4 | 9 | 3 | 6 |
| Beija-Flor C4 x Synthetic UFV | 8 | 4 | 4 |
| UFV-MP2 x UFV-MP5 | 22 | 22 | 0 |
| Viçosa-C4 x UFV-MP2 | 9 | 6 | 3 |
| Viçosa C4 x UFV-MP4 | 21 | 14 | 7 |
| Viçosa C4 x UFV-MP5 | 4 | 3 | 1 |
| Synthetic UFV x UFV-MP1 | 46 | 46 | 0 |
| 2B710PW | 59 | 47 | 12 |
| MG580PW | 54 | 44 | 10 |
| ES481PW | 20 | 13 | 7 |
| Feroz | 22 | 14 | 8 |
| AGS Lavrador OP | 10 | 8 | 2 |
| Total | 677 | 459 | 218 |

**Supplementary Table 4**. Screening by the ploidy level.

| Germplasm | Seedlings | Haploids | False positive |
| --- | --- | --- | --- |
| 18-176-5 | 108 | 0 | 108 |
| 20-2009 | 9 | 8 | 1 |
| 20-2010 | 63 | 6 | 57 |
| 20-2064 | 30 | 15 | 15 |
| 20-2024 | 29 | 14 | 15 |
| UFV-MP1 | 4 | 2 | 2 |
| UFV-MP5 | 5 | 1 | 4 |
| Viçosa C4 | 54 | 3 | 51 |
| Beija-Flor C4 | 7 | 0 | 7 |
| Synthetic UFV | 54 | 4 | 50 |
| Viçosa-C4 x UFV-MP2 | 9 | 2 | 7 |
| Viçosa C4 x UFV-MP4 | 21 | 5 | 16 |
| Synthetic UFV x UFV-MP1 | 46 | 0 | 46 |
| MG580PW | 54 | 18 | 36 |
| Total | 677 | 78 | 415 |
